# The History and Capabilities of Herbal Simples

**Published:** 1890-07-26

**Authors:** W. T. Fernie


### The History and Capabilities of Herbal Simples.

By W. T. Fernie, M.D.

*(Continued from page 228.)*

### XVI.—CAMOMILE.

"The more you tread on camomile the faster it grows," says an old English proverb. And yet no simple in the whole catalogue of medicines is possessed of a quality more friendly and beneficial to the intestines than "camomile flowers." So I doff my hat to the comforting panacea, and shall treat the familiar old plant with more respect than to trample it down and pass it by unheeded. This herb was well known to the Greeks, who thought it had a smell like that of apples; and therefore they named it the Earth Apple, from two of their words—Kamai, on the ground, and Melon, an apple. The Spaniards call it Manzanilla, from "a little apple," and they give the same name to one of their best and lightest sherries, which is flavoured with this plant. The flowers, or blows, of the camomile belong to the daisy genus, having an outer fringe of white ray florets, with a central yellow disc, in which lies the chief medicinal virtue of the plant. In the cultivated camomile the white petals increase in size, whilst the yellow centre diminishes; thus it is that the curative properties of the wild camomile are more powerful. This true wild camomile is a frequent flower amid the grass of our waysides and fields. It is to be distinguished from the bitter camomile, or Corn Feverfew, which possesses the virtues of the true camomile in only quite an inferior degree, and grows erect, with several flowers at a level on the same stalk. The true camomile grows prostrate, and produces but one flower, with a convex yellow disc from each stem, whilst its leaves are divided into hairlike segments.

Who has not found, at one time or another, soothing ease from hot fomentation of camomile flowers, whether for a stomach ache, or for a swollen face, or some other minor ailment to which our flesh is heir? Every part of the plant (Anthemis nobilis) evolves a strong fragrant odour, and is bitter. It contains a dark blue, or dark green essential oil, which is the chemical source of its activity. This oil is a mixture of ethers, among which camomilline, or the valerianate of butyl, predominates. Medicinally it serves to lower reflected nervous excitibility; so it is very useful for some spasmodic coughs, for nervous colic of the bowels, and for many neuralgic pains arising from impaired digestion. The oil may be given in doses of from two to six drops on sugar, or in milk. It should be green, or blue, of good quality, and not faded to yellow. Bags, or legs of stockings, loosely stuffed with the flowers, and steeped thoroughly in boiling water before being applied, are most useful for relieving inflammatory neuralgic troubles and swellings. The camomile is likewise reputed to be a plant physician; for, nothing contributes so much to the health of a garden as a number of camomile plants dispersed through it. Singularly enough, if any other plant is drooping, and apparently dying, in nine cases out of ten it will recover if you place a plant of camomile near it. This, and the feverfew, or bitter camomile, are very obnoxious to flies and mosquitoes. By infusing the powdered leaves in spirit and water, and applying the same as a wash to the face, hands, or any exposed part of the body, a traveller may be effectually protected from these petty virulent foes, which an old version of our Bible quaintly terms "the pestilence that walketh in the darkness, and the bug that destroyeth at noonday."

Camomile tea is an excellent stomachic when taken in moderate doses of one or two tablespoonfuls; but a teacupful will cause vomiting. It may be made by pouring half a pint of boiling water on half an ounce of the dried flower heads, and letting this stand for fifteen minutes. A special tincture is made of "camomilla," from the bitter camomile—matricaria—which, when given in small doses of three or four drops every hour, will signally relieve severe neuralgic pains, particularly if they are aggravated at night. Likewise this remedy will quickly cure restlessness and fretfulness in children from teething, and a refusal to be soothed save when being carried about. The name matricaria is derived from "mater cara"—beloved mother—because the plant is dedicated to St. Anne, the reputed mother of the Virgin. It is also called Mayweed, from "may," a maiden, because used for the complaints of young women. So highly did the Egyptians think of the camomile that they dedicated this herb to the sun. Dr. Schall declares that camomile is not only an effectual preventive of nightmare, but the sole certain remedy for this complaint. As a carminative clyster for tiresome flatulence it has been found eminently beneficial to employ camomile flowers boiled in tripe broth, strained through a cloth, and with a few drops of oil of aniseed added to the decoction. Falstaff says in Henry IV., "Though the camomile the more it is trodden on the faster it grows; yet youth the more it is wasted the sooner it wears." For coarse feeders and drunkards camomile is peculiarly appropriate. Its infusion will cut short an attack of delirium tremens in the early stage. Gerarde found the oil of the flowers a remedy against all weariness. Quaint old Culpeper repeats the statement that the Egyptians dedicated the camomile to the sun because it cured agues; and he adds, "They were like enough to do it, for they were the arrantest apes in their religion I ever read of."

